# Optimal Initial Time Point of Local Radiotherapy for Unresectable Lung Adenocarcinoma: A Retrospective Analysis on Overall Arrangement of Local Radiotherapy in Advanced Lung Adenocarcinoma

**DOI:** 10.3389/fonc.2022.793190

**Published:** 2022-02-10

**Authors:** Xinge Li, Jie Wang, Xu Chang, Zhenhua Gao, Feifei Teng, Xue Meng, Jinming Yu

**Affiliations:** ^1^ Department of Radiation Oncology, The First Hospital of China Medical University, Shenyang, China; ^2^ Department of Radiation Oncology, Shandong Cancer Hospital and Institute, Shandong First Medical University and Shandong Academy of Medical Sciences, Jinan, China; ^3^ Cheeloo College of Medicine, Shandong University, Jinan, China

**Keywords:** radiotherapy, local radiotherapy, optimal time point, unresectable lung adenocarcinoma, non-small cell lung cancer

## Abstract

Local radiotherapy (LRT) is reported to be of survival benefit for advanced non-small cell lung cancer (NSCLC) in accumulating evidence, but research on the optimal initial time point remains scarce. This IRB-approved retrospective analysis identified patients diagnosed with stage IIIb–IV unresectable lung adenocarcinoma who initiated front-line LRT at our institution between 2017 and 2020. The receiver operating characteristic (ROC) curve analyses were used to cut off the initial time of LRT (before and beyond 53 days). Patients were divided into two groups: one early to initiate radiotherapy group (≤53 days, EAR group) and one deferred radiotherapy group (>53 days, DEF group). The Kaplan–Meier method was used to estimate time-to-event endpoints; the Cox proportional hazard model was used to find out predictors of progression-free survival (PFS) and overall survival (OS). A total of 265 patients with a median age of 57 were enrolled. The median follow-up time was 26.4 months (ranging from 2.2 to 69.7 months). The mOS was 38.6 months and mPFS was 12.7 months. Age >60, bone and brain metastases, multisite metastases, and EGFR 19 mutation were independent predictors associated with OS. Early initiation of local radiotherapy within 53 days after diagnosis resulted in better PFS, but not in OS. A better OS was observed in patients with bone metastasis who underwent local radiotherapy initiated within 53 days.

## Introduction

Lung cancer ranks only second to breast cancer in incidence and the top above any other cancer in mortality around the world, accounting for 18% of cancer deaths, according to GLOBOCAN 2020 data. Traditional surgical resection is the treatment of choice for patients with operable non-small-cell lung cancer (NSCLC) (1, 2). Patients with stage IIIb–IV unresectable NSCLC are seeing a dismal prognosis (2–5). Nevertheless, the springing up of molecular understanding and the development of molecular detection techniques within the last decade have refreshed the management for patients with NSCLC harboring oncogenic mutations (6, 7). Targeted therapeutic strategies, in addition to cytotoxic systemic chemotherapy over the past decade, have fostered a rising shift of survival benefit (8, 9).

Radiotherapy, along with other local ablative strategies, is regarded as efficient means to alleviating symptoms as well as promoting local lesion control in advanced NSCLC (10, 11). The emerging conception of oligometastases brought us more consideration for management of patients with limited number of metastatic lesions (12–14). Several remarkable prospective randomized trials have demonstrated the profit from local consolidative intervention not merely in local control but also in survival outcome for patients with advanced NSCLC. The first multi-institutional randomized trial led by the MD Anderson Cancer Center demonstrated the progression-free survival and overall survival benefit in local consolidative therapy compared with standard maintenance therapy (15, 16). Another randomized trial contemporaneously led by investigators at the University of Texas revealed that stereotactic ablative radiotherapy (SABR) in addition to induction systemic therapy and maintenance therapy prolonged progression-free survival (PFS) from 3.5 to 9.7 months (17). The third randomized trial that showed a considerable improvement in survival with the implementation of SABR for patients with oligometastatic disease was the SABR-COMET trial, of which NSCLC patients took up approximately 18% patients enrolled (18).

Even though reasonable trials indicate the impressive benefit that may be obtained through the implementation of local interventions, debate on its optimal timing remains scarce. Previous studies focused mainly on intervention in the process of consolidation section instead of earlier phases of treatment regimen during which time a little diversification may result in a large discrepancy later on. Thus, we hypothesized that earlier initiation of local radiotherapy for patients with stage IV NSCLC may offer better survival benefit. To address this hypothesis, we investigate the initial timing of radiation therapy and survival outcome of patients with stage IIIb–IV unresectable lung adenocarcinoma, with or without oncogenic mutations.

## Materials and Methods

### Patients

**Figure 1:**
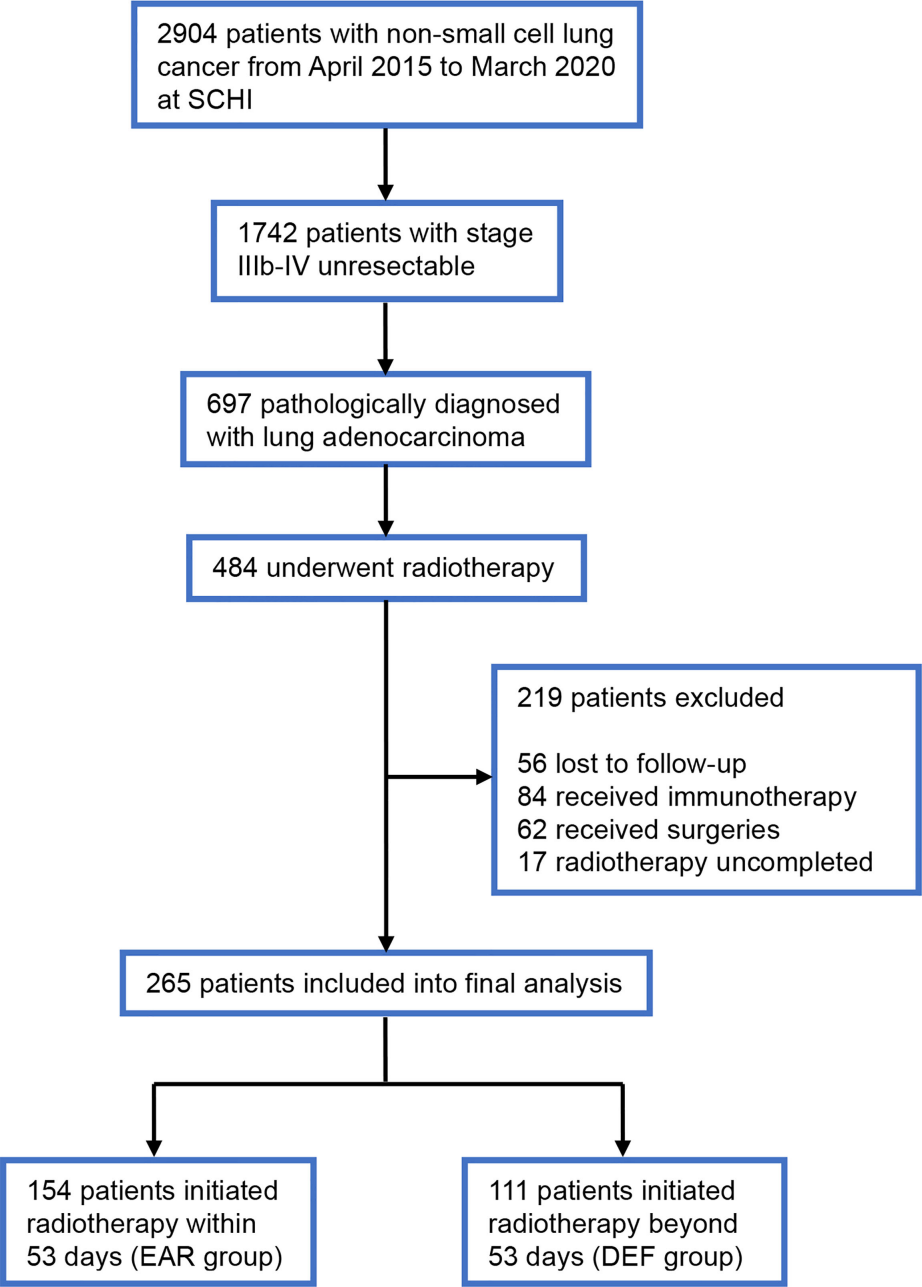
Flowchart of the patient cohort. SCHI, Shandong Cancer Hospital and Institute; EAR, early to initiate radiotherapy group; DEF, deferred radiotherapy group.

We conducted a retrospective study and reviewed the medical records of patients diagnosed with stage IIIb–IV unresectable lung adenocarcinoma at Shandong Cancer Hospital and Institute from January 2017 to March 2020. Patients eligible for this analysis should meet the following criteria: (1) stages IIIb–IV (according to the 7th edition of the American Joint Committee on Cancer staging system) pathologically diagnosed with lung adenocarcinoma; (2) treatment-naïve when at first diagnosis; (3) received local radiation therapy during front-line treatment; (4) aged 18 years or older, with a Karnofsky Performance Status (KPS) of 70 or higher; and (5) has adequate follow-up data. Patients were excluded when (1) with a history of non-standard treatment of immunotherapy; (2) with a history of local interventions other than radiotherapy; and (3) full dose and course of radiotherapy was uncompleted. Patient clinical data, including sex, age at diagnosis, time of diagnosis, Karnofsky Performance Status, status of T, N, M stages, metastasis sites, status of oncogenic mutations, systemic treatment regimen, initial time of radiotherapy, status and time of progression, and status and time of death were collected and collated from medical records (**Figure 1**). Data were cut off by August 22, 2021. This study was approved by the institutional review board of Shandong Cancer Hospital and was conducted in accordance with the Declaration of Helsinki. Informed consent to access the electronic medical record was obtained from each participant.

### Time Division

**Figure 2:**
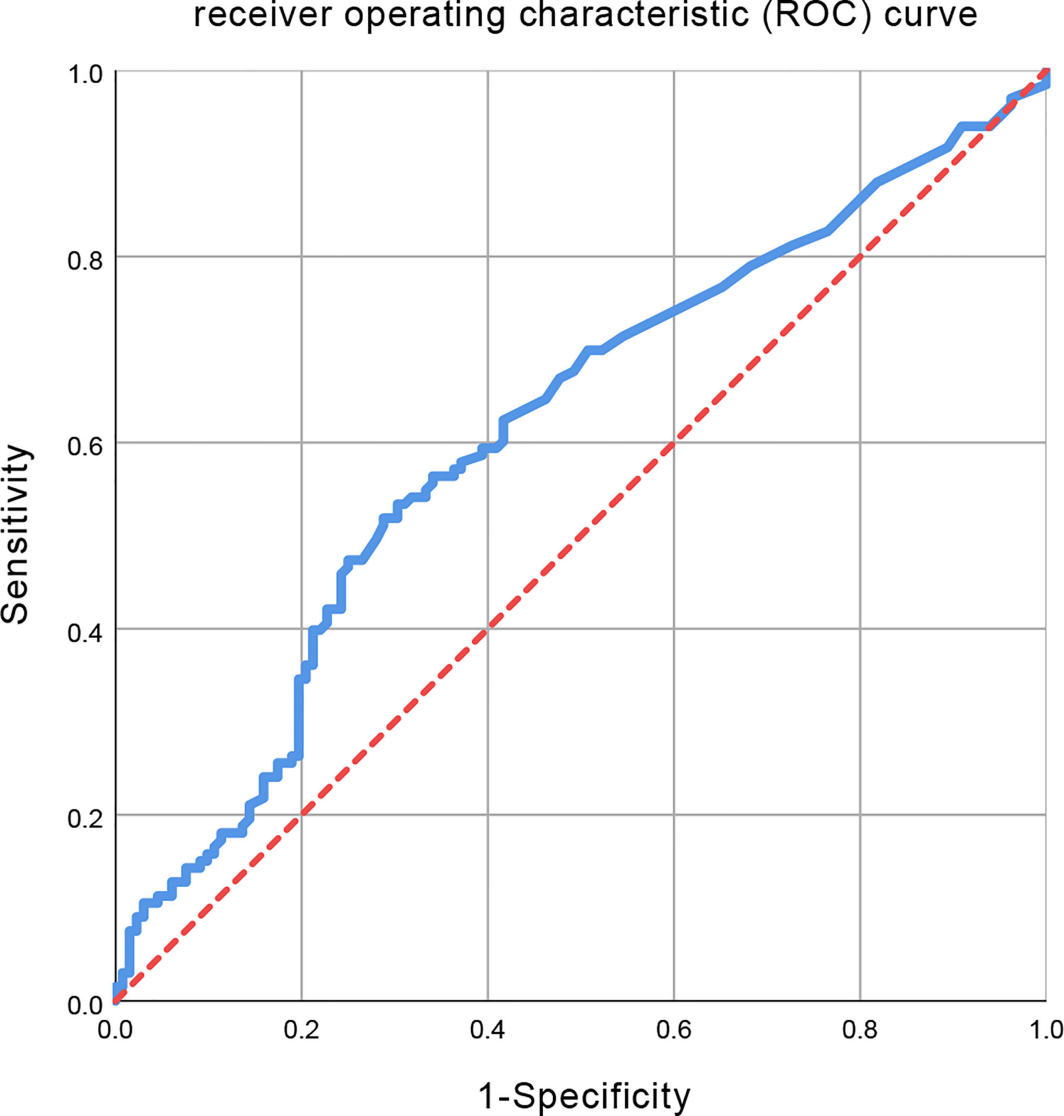
Receiver operating characteristic (ROC) curve for all initiation time of radiotherapy. The area under the curve (AUC) was 0.613.

The initiation time of radiotherapy was calculated as the time interval from diagnosis to the initiation of radiotherapy. Logistic regression analyses were used to assess the initiation time of radiotherapy associated with disease progression. The receiver operating characteristic (ROC) curve analyses were used for the identification of the cutoff values of the time interval. The ROC curve with an area under curve (AUC) of 0.613 was obtained (**Figure 2**). The optimal cutoff values were determined using Youden’s index which was calculated as the maximum value of the formula: sensitivity – (1 – specificity). Subsequently, a Youden’s index of 0.217 and the cutoff value of 53 days were obtained. Then, the patients were divided into two groups based on this cutoff value: one early to initiate radiotherapy group (≤53 days, EAR group) and one deferred radiotherapy group (>53 days, DEF group).

### Systemic Medication Regimen

Definitive systemic therapy has constantly been the cornerstone of the treatment paradigm for unresectable locally advanced or metastatic NSCLC. In lung adenocarcinoma, patients harboring oncogenic mutations can benefit from tyrosine kinase inhibitor (TKI) therapy. Others received conventional cytotoxic platinum-based chemotherapy. In real-world practice, next-generation sequencing can be time-consuming. Some of these patients thereby received cytotoxic chemotherapy ahead of TKIs in order to get timely treatment during their wait for genomic testing reports. This part of patients was grouped and classified into combination of “chemotherapy and targeted therapy” group in our analysis. All systemic therapy was administered using standard-of-care first-line regimens, with the choice of specific medications at the discretion of the oncologist. Patients who have received second-line or above systemic medication were excluded from our analysis. A fraction of patients received immunotherapy was also excluded, to avoid confounding that may be caused by inherent heterogeneities.

### Procedure of Radiotherapy

All radiotherapy patients received was external beams *via* intensity-modulated radiation therapy (IMRT). All patients received standard prescription dose according to their respective stages. Patients enrolled to our analysis were treated using the same treatment planning system, standard procedures, and radiation dose constraints for organs at risk. Each individual gross tumor volume (GTV) was contoured referring to CT, MRI, and FDG-PET reports, then expanded up to 5 mm to clinical target volume (CTV), then another 5 mm to planning target volume (PTV). The prescription dose and fraction mode for different sites were determined by the treating radiologist, ranging from a palliative dose to a definite one based on tumor conditions. If multiple lesions were existing in close proximity, effort was made to treat them with one dose and fractionation. All radiation treatment plans were reviewed by a board consisting of a radiologist, radiographer, and medical physicist based on CB-CHOP standard before implementation. For patients who received more than one course of radiotherapy, only the initiation time of the first course was included into our analysis.

### Follow-Up

Tumor stage was assessed by systemic imaging features: either contrast-enhanced computed tomography (CT) for the brain, chest, abdomen, and bone, or positron emission tomography/computed tomography (PET-CT) with brain magnetic resonance imaging (MRI). Patients’ response was measured by imaging technologies mentioned above and characterized by Response Evaluation Criteria in Solid Tumors (RECIST) for both the primary tumor and the metastatic sites of disease. Patients’ progression or survival conditions were followed up by telephone number extracted from medical records.

### Statistical Analysis

Progression-free survival (PFS) was calculated as the time period from the date of treatment initiation to the date of disease progression, including progression *in situ*, in metastasis and new onset of metastatic site. Overall survival (OS) was calculated as the time period from the date of diagnosis to the date of death of any cause or the date of data cutoff. Descriptive statistics were used to summarize baseline characteristics. The chi-square test and Fisher’s exact test were used to compare categorical variables. The Kaplan–Meier method and log-rank tests were used for OS and PFS analyses as well as comparison of different groups. p < 0.05 was defined as statistically significant. All statistical analyses were performed using SPSS V26.0 (IBM Corporation, Armonk, NY, USA).

## Results

### Patient Characteristics

A total of 265 patients with stage IIIb–IV unresectable lung adenocarcinoma who underwent front-line full-course radiotherapy during the study period were enrolled. The patient characteristics are presented in **Table 1**. The majority of patients (142, 53.6%) were male. The median age was 57 years (range from 24 to 78 years), 165 patients (62.3%) aged under 60 years old and 100 (37.7%) above. Patients with a KPS score above 90 or 80 accounts for 46.8% and 49.1%, respectively, of all patients. Patients with unresectable locally advanced lung adenocarcinoma account for 17.7% of all patients enrolled. The most common site of metastasis was brain (61,23%) of all 265 patients, followed by multisite metastases (57,21.5%), no metastasis, and bone metastasis respectively (50,18.9%). Metastasis sites that were relatively infrequent were classified into other-site group (12,4.5%). Regarding oncogenic mutations, 38.1% patients bore no mutations, 25.7% patients bore EGFR 21, 19.2% EGFR 19, 3.8% ALK, and 13.2% other rare mutations. 154 patients who initiated radiotherapy within 53 days were allocated into the EAR group, and 111 patients that initiated radiotherapy beyond 53 days were allocated into the DEF group. Most of the patients received TKIs combined with chemotherapy in both EAR (56,36.4%) and DEF (36,32.4%) groups, as well as in all patients (92,34.7%). The stages of T and N and types of systemic regimen used are shown in **Table 1**.

**Table 1:** Baseline patient characteristics.

Characteristics	EAR (≤53 days)	DEF (>53 days)	Total	*p*-value
	N (%)	N (%)	N (%)	
**Sex**				
Male	72 (46.8)	70 (63.1)	142 (53.6)	0.009
Female	82 (53.2)	41 (36.9)	123 (46.4)	
**Age**				
≤60	94 (61)	71 (64)	165 (62.3)	0.7
>60	60 (39)	40 (36)	100 (37.7)	
**KPS**				
≥90	66 (42.9)	58 (52.3)	124 (46.8)	0.117
≥80	78 (50.6)	52 (46.8)	130 (49.1)	
≥70	10 (6.5)	1 (0.9)	11 (4.2)	
**T**				
1	37 (24)	18 (16.2)	55 (20.8)	
2	62 (40.3)	49 (44.1)	111 (41.9)	0.411
3	20 (13)	19 (17.1)	39 (14.7)	
4	35 (22.7)	25 (22.5)	60 (22.6)	
**N**				
0	30 (19.5)	19 (17.1)	49 (18.5)	
1	6 (3.9)	5 (4.5)	11 (4.2)	0.513
2	62 (40.3)	37 (33.3)	99 (37.4)	
3	56 (36.4	50 (45)	106 (40)	
**M**				
0	9 (5.8)	38 (34.2)	47 (17.7)	<0.001
1	145 (94.2)	73 (65.8)	218 (82.3)	
**Metastasis sites**				
None	10 (6.5)	40 (36)	50 (18.9)	
Brain	47 (30.5)	14 (12.6)	61 (23)	
Bone	33 (21.4)	17 (15.3)	50 (18.9)	<0.001
Bilateral pulmonary	2 (1.3)	9 (8.1)	11 (4.2)	
Bone and brain	20 (13)	4 (3.6)	24 (9.1)	
Other sites	0 (0)	12 (10.8)	12 (4.5)	
Multisites	42 (27.3)	15 (13.5	57 (21.5)	
**Oncogenic mutation**				
None	49 (31.8)	52 (46.8)	101 (38.1)	
EGFR 19	30 (19.5)	21 (18.9)	51 (19.2)	0.084
21	46 (29.9)	22 (19.8)	68 (25.7)	
ALK	5 (3.2)	5 (4.5)	10 (3.8)	
Others	24 (15.5)	11 (9.9)	35 (13.2)	
**Systemic medication**				
Chemo	28 (18.2)	36 (32.4)	64 (24.2)	
Bev and chemo	34 (22.1)	27 (24.3)	61 (23)	0.09
TKIs	36 (23.4)	12 (10.8)	48 (18.1)	
TKIs and chemo	56 (36.4)	36 (32.4	92 (34.7)	

EAR, early to initiate radiotherapy group; DEF, deferred radiotherapy group; Bev, bevacizumab; chemo, chemotherapy; TKIs, tyrosine kinase inhibitors.

### Survival Outcome

**Figure 3:**
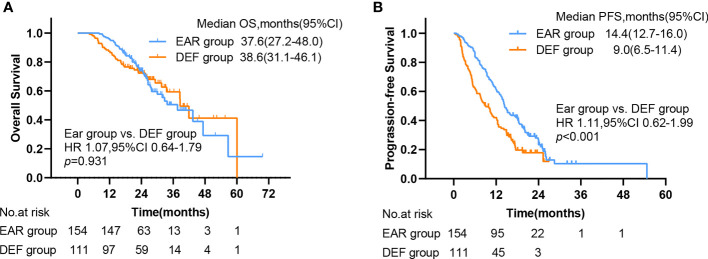
Kaplan–Meier plots of **(A)** OS and **(B)** PFS in EAR and DEF, with numbers at risk shown below the graph. PFS, progression-free survival; OS, overall survival; CI, confidence interval, EAR, early to initiate radiotherapy group (≤53 days); DEF, deferred radiotherapy group (>53 days).

The median follow-up time was 26.4 months (ranging from 2.2 to 69.7 months). 205 patients had disease progression (118 in the EAR group, 87 in the DEF group), and 172 patients were alive at the last follow-up (101 in the EAR group, 71 in the DEF group). The median OS (mOS) and median PFS (mPFS) for the cohort was 38.6 months (95% CI, 31.8–45.4) and 12.7 months (95% CI, 11.0–14.4), respectively. As shown in **Figure 3A**, the mOS for the EAR group and DEF group were 37.6 months (95% CI, 27.2–48) and 38.6 months (95% CI, 31.1–46.1), respectively. No significance was observed when comparing these two groups (HR 1.07, 95% CI 0.64–1.79, p = 0.931). The mPFS for the EAR group and DEF group is shown in **Figure 3B**. The mPFS for the EAR group was 14.4 months (95% CI, 12.7–16.0) and 9 months (95% CI, 6.5–11.4) for the DEF group. A remarkable significance in PFS was observed in the EAR group compared with the DEF group (HR 1.11, 95% CI 0.62–1.99, p < 0.001).

**Figure 4:**
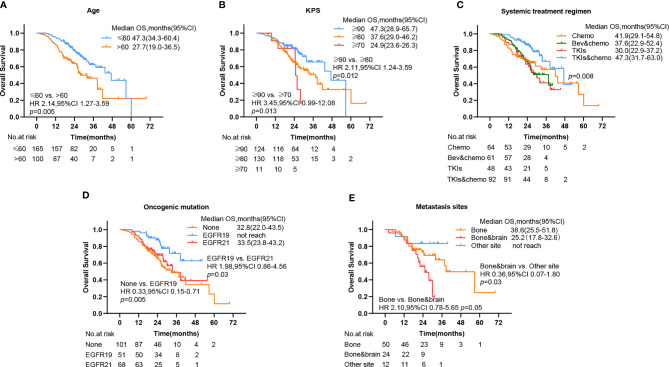
Kaplan–Meier plots show percent overall survival categorized by **(A)** age, **(B)** KPS, **(C)** systemic treatment regimen, **(D)** oncogenic mutation, and **(E)** metastasis sites, with numbers at risk shown below the graph. PFS, progression-free survival; OS, overall survival, CI, confidence interval, Bev, bevacizumab; chemo, chemotherapy; TKIs, tyrosine kinase inhibitors.

Of note, significant survival differences were noticed in other grouping methods in addition to different initial times of radiotherapy. The mOS for patients aged under 60 was 47.3 (95% CI, 34.3–60.4) and 27.7 (95% CI, 19.0–36.5) for patients aged above 60, with a significant better OS of the former group than the latter (HR 2.14, 95% CI 1.27–3.59, p = 0.005) (**Figure 4A**). The mOS for patients with a KPS score above 90, 80, and 70 was 47.3 (95% CI, 28.9–65.7), 37.6 (95% CI, 29.0–46.2), and 24.9 (95% CI, 23.6–26.3), respectively. The OS for the former group was significantly better than the latter two (**Figure 4B**). The mOS for patients who received chemotherapy, bevacizumab plus chemotherapy, TKIs, and TKIs plus chemotherapy was 41.9 (95% CI, 29.1–54.8), 37.6 (95% CI, 22.9–52.4), 30.0 (95% CI, 22.9–37.2), and 47.3 (95% CI, 31.7–63.0), respectively. The combination of the TKIs and chemotherapy group showed significant better OS compared with other groups (p = 0.008) (**Figure 4C**). The OS for patients who bore no oncogenic mutation, EGFR 19, and EGFR 21 was 32.8 (95% CI, 22.0–43.5), not reached, and 33.5 (95% CI, 23.8–43.2), respectively. The OS for patients with EGFR 19 mutation was significantly better than that of others (**Figure 4D**). The OS for patients with bone and brain metastases (25.2, 95% CI 17.8–32.6) was significantly worse than that of patients with other metastases (**Figure 4E**).

**Figure 5:**
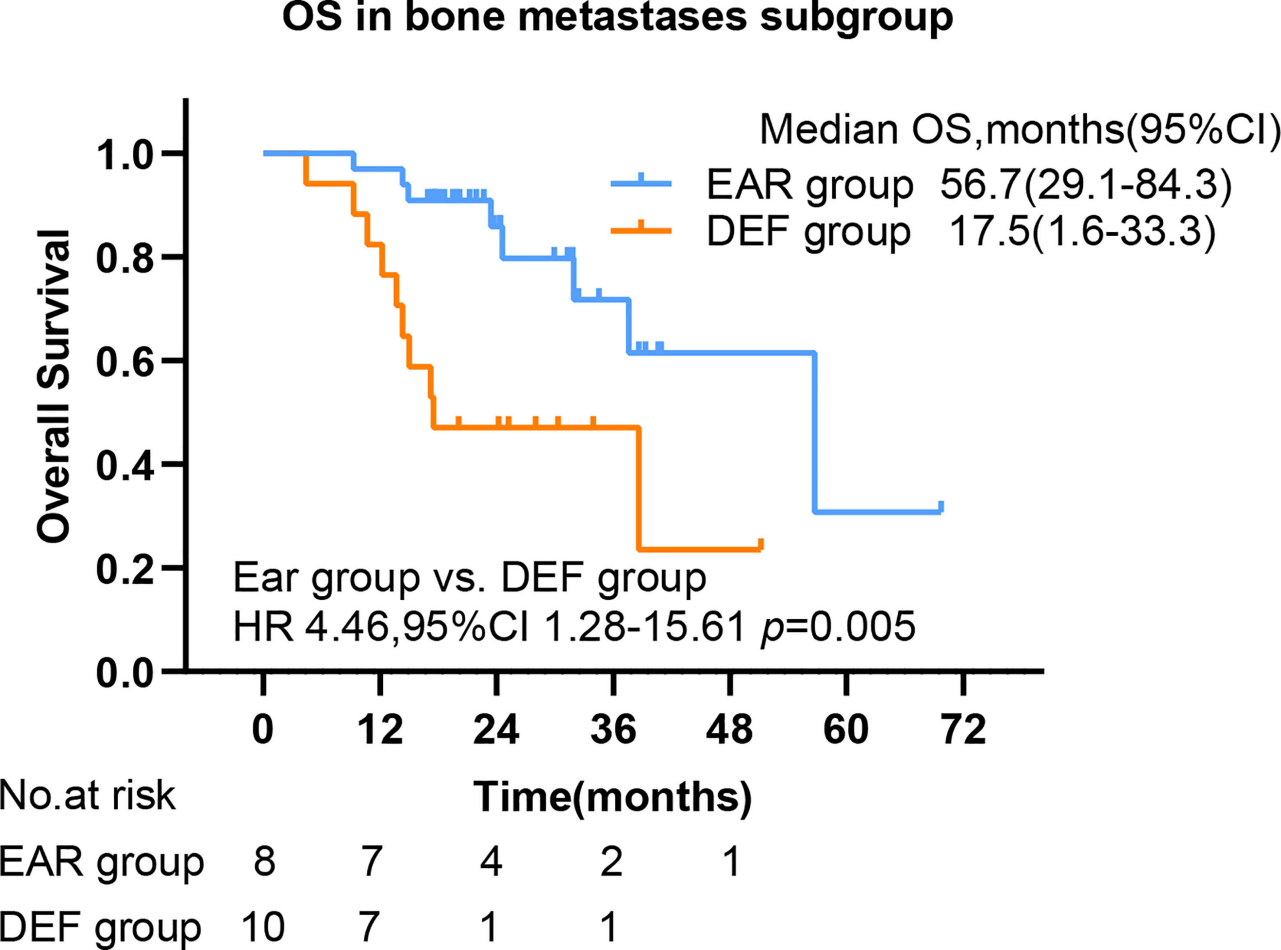
The OS by bone metastasis. PFS, progression-free survival; OS, overall survival; CI, confidence interval, EAR, early to initiate radiotherapy group (≤53 days); DEF, deferred radiotherapy group (>53 days).

**Figure 6:**
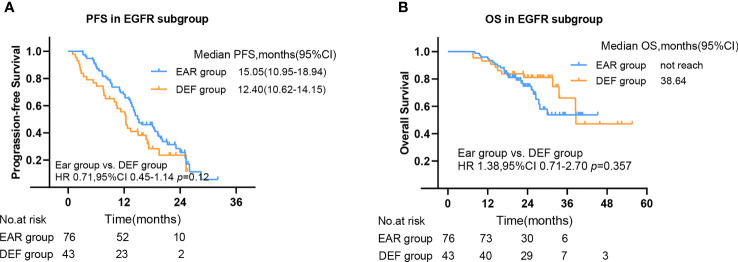
The PFS **(A)** and OS **(B)** in EGFR mutant subgroup. PFS, progression-free survival; OS, overall survival; CI, confidence interval, EAR, early to initiate radiotherapy group (≤53 days); DEF, deferred radiotherapy group (>53 days).

To further investigate the beneficial populations from early initial radiotherapy, we subdivided patients by metastasis sites. Interestingly, the mOS were significantly improved with the use of early initial radiotherapy than deferred radiotherapy for patients with bone metastasis (56.7 versus 17.5 months, HR 4.46, 95% CI 1.28–15.61, p = 0.005) (**Figure 5**). Patients with EGFR mutations occupy nearly half of all patients. Subgroup analysis was added to the EGFR patient cohort. No significance was observed in PFS in the EAR group and DEF group (HR 0.71, 95% CI 0.45–1.14 p = 0.12), or in OS (HR 1.38, 95% CI 0.71–2.70 p = 0.357) (**Figures 6A, B** **)**.

### Univariate and Multivariate Analyses on PFS and OS

On univariable analysis, T2, EGFR 19 mutation, systemic medication, and initial time of radiotherapy were associated with PFS (p < 0.1). On multivariate analysis, KPS ≥ 80 (HR 1.30, 95% CI 0.95–1.76, p = 0.09), T2 (HR 1.46, 95% CI 0.96–2.22, p = 0.07), and initial time of radiotherapy (HR 1.92, 95% CI 1.39–2.66, p < 0.001) were independent predictors associated with decreased PFS. The systemic treatment regimen of combination of TKIs and chemotherapy (HR 0.54, 95% CI 0.32–0.92, p = 0.02) was the independent predictor associated with favorable PFS (**Table 2**).

**Table 2:** Univariable and multivariable analyses of covariables associated with PFS.

Variable	Univariable analysis			Multivariable analysis		
	HR	95% CI	*p*-value	HR	95% CI	*p*-value
**Sex**						
Male vs. female	0.82	0.62–1.08	0.15			
**Age**						
≤60 vs. >60	0.97	0.73–1.29	0.85	0.79	0.58–1.08	0.14
**KPS**			0.19			0.13
≥90						
≥80	1.26	0.95–1.67	0.11	1.30	0.95–1.76	0.09
≥70	1.48	0.77–2.85	0.24	1.68	0.84–3.34	0.13
**T**			0.25			0.30
1						
2	1.42	0.96–2.10	0.07	1.46	0.96–2.22	0.07
3	1.38	0.86–2.23	0.17	1.37	0.83–2.28	0.21
4	1.13	0.73–1.75	0.57	1.18	0.73–1.90	0.49
**N**			0.88			
0						
1	1.02	0.47–2.19	0.95			
2	1.00	0.68–1.48	0.96			
3	0.89	0.61–1.31	0.58			
**M**						
0 vs. 1	0.87	0.61–1.26	0.48			
**Metastasis sites**			0.99			0.82
None						
Brain	0.92	0.60–1.42	0.72	1.23	0.75–2.03	0.40
Bone	0.89	0.57–1.40	0.63	1.24	0.73–2.08	0.41
Bilateral pulmonary	1.00	0.46–2.14	1.00	0.88	0.40–1.96	0.76
Bone and brain	0.91	0.53–1.57	0.74	1.40	0.76–2.59	0.27
Other sites	1.11	0.53–2.29	0.78	0.90	0.42–1.91	0.78
Multi sites	0.95	0.61–1.47	0.81	1.35	0.83–2.21	0.22
**Oncogenic mutation**			0.08			0.44
None						
EGFR 19	0.58	0.39–0.86	0.007	0.67	0.38–1.19	0.17
21	0.75	0.52–1.06	0.10	1.01	0.58–1.75	0.96
ALK	0.68	0.33–1.41	0.30	0.76	0.33–1.74	0.52
Others	0.72	0.46–1.12	0.15	0.92	0.51–1.64	0.78
**Systemic medication**			0.001			0.05
Chemo						
Bev and chemo	0.97	0.66–1.43	0.90	0.90	0.59–1.37	0.63
TKIs	0.83	0.55–1.24	0.37	0.88	0.49–1.57	0.67
TKIs and chemo	0.51	0.35–0.74	<0.001	0.54	0.32–0.92	0.02
**Time of radiotherapy**						
EAR vs. DEF	1.66	1.25–2.21	<0.001	1.92	1.39–2.66	<0.001

HR, hazard ratio; CI, confidence interval; Bev, bevacizumab; chemo, chemotherapy; TKIs, tyrosine kinase inhibitors; EAR, early to initiate radiotherapy group; DEF, deferred radiotherapy group.

On univariable analysis, age >60, KPS ≥ 80, KPS ≥ 70, bone and brain metastases, multisite metastases, EGFR 19 mutation, and systemic treatment regimen of combination of TKIs and chemotherapy were associated with OS (p < 0.1). On multivariate analysis, age >60 (HR 1.48, 95% CI 0.94–2.31, p = 0.08), bone and brain metastases (HR 2.77, 95% CI 1.16–6.6, p = 0.02), and multisite metastases (HR 2.06, 95% CI 1.01–4.21, p = 0.04) were independent predictors associated with decreased OS. EGFR 19 mutation was an independent predictor associated with favorable OS (**Table 3**).

**Table 3:** Univariable and multivariable analyses of covariables associated with OS.

Variable	Univariable analysis			Multivariable analysis		
	HR	95% CI	*p*-value	HR	95% CI	*p*-value
**Sex**						
Male vs. female	0.82	0.54–1.24	0.36			
**Age**						
≤60 vs. >60	1.97	1.31–2.96	0.001	1.48	0.94–2.31	0.08
**KPS**			0.01			0.08
≥90						
≥80	1.73	1.12–2.69	0.01	1.46	0.92–2.31	0.10
≥70	2.76	1.14–6.63	0.02	2.53	0.99–6.42	0.05
**T**			0.29			
1						
2	1.58	0.87–2.88	0.12			
3	1.04	0.47–2.29	0.92			
4	1.14	0.57–2.27	0.69			
**N**			0.95			
0						
1	1.22	0.41–3.65	0.71			
2	1.13	0.63–2.01	0.67			
3	1.02	0.57–1.82	0.94			
**M**						
0 vs. 1	1.62	0.89–2.92	0.10			
**Metastasis sites**			0.06			0.16
None						
Brain	1.56	0.76–3.18	0.22	1.55	0.73–3.30	0.25
Bone	1.47	0.71–3.00	0.29	1.28	0.59–2.76	0.52
Bilateral pulmonary	1.51	0.49–4.67	0.47	1.49	0.46–4.75	0.50
Bone and brain	3.23	1.47–7.06	0.003	2.77	1.16–6.60	0.02
Other sites	0.71	0.16–3.17	0.65	0.68	0.15–3.16	0.63
Multi sites	2.18	1.10–4.33	0.02	2.06	1.01–4.21	0.04
**Oncogenic mutation**			0.06			0.22
None						
EGFR 19	0.39	0.20–0.77	0.007	0.39	0.16–0.94	0.03
21	0.86	0.52–1.42	0.57	0.78	0.34–1.78	0.56
ALK	0.46	0.14–1.49	0.19	0.49	0.13–1.89	0.30
Others	0.66	0.32–1.37	0.27	0.80	0.32–2.00	0.63
**Systemic medication**			0.01			0.41
Chemo						
Bev and chemo	1.19	0.68–2.08	0.54	1.05	0.57–1.94	0.86
TKIs	1.31	0.73–2.35	0.35	1.31	0.54–3.14	0.54
TKIs and chemo	0.49	0.27–0.91	0.02	0.74	0.32–1.70	0.46
**Time of radiotherapy**						
EAR vs. DEF	0.98	0.65–1.48	0.93			

HR, hazard ratio; CI, confidence interval; Bev, bevacizumab; chemo, chemotherapy; TKIs, tyrosine kinase inhibitors; EAR, early to initiate radiotherapy group; DEF, deferred radiotherapy group.

### Toxicity

The most common toxicity during the treatment course was hematologic toxicity for both EAR (43,27.9%) and DEF (33,29.7%) groups, which was mostly grade 2. The occurrence of gastrointestinal toxicity for two groups was 7.7% and 9.0%. The radiotherapy-related adverse events were mainly pneumonitis, esophagitis, and dermatitis. None of these toxicities mentioned above were statistically significant between two groups. Interestingly, the occurrence of radiation pneumonitis in the DEF group was statistically higher than that in the EAR group in our analysis (p = 0.01) (**Table 4**). All adverse events were tolerable when timely treated.

**Table 4:** Toxicity profile for the EAR and DEF groups.

Toxicity Outcomes	EAR group N = 154 (58.1%)	DEF group N = 111 (41.9%)	*p*-value
Hematologic toxicity			
Grade 1	14 (9.1%)	8 (7.2%)	0.58
Grade 2	22 (14.3%)	19 (17.1%)	0.52
Grade 3	6 (3.9%)	5 (4.5%)	0.80
Grade 4	1 (0.6%)	1 (0.9%)	0.81
Gastrointestinal toxicity			
Grade 1	8 (5.2%)	8 (7.2%)	0.49
Grade 2	3 (1.9%)	2 (1.8%)	0.93
Grade 3	1 (0.6%)	0 (0)	0.39
Liver dysfunction	4 (2.6%)	5 (4.5%)	0.40
Skin rash	2 (1.3%)	4 (3.6%)	0.21
Diarrhea	1 (0.6%)	0 (0)	0.39
Radiation pneumonitis	3 (1.9%)	9 (8.1%)	0.01
Radiation esophagitis	1 (0.6%)	4 (3.6%)	0.08
Radiation dermatitis	1 (0.6%)	0 (0)	0.39

## Discussion

Lung adenocarcinoma, especially with activating EGFR mutations, has better survival outcome among patients with unresectable NSCLC (19–21). Therefore, exploring an optimal implementation time point of local radiotherapy is meant for this group of patients. This analysis sought to find out the optimal initial timing of radiotherapy in unresectable stage IIIb–IV lung adenocarcinoma. To our knowledge, the present study is the largest one to statistically investigate the optimal initial timing of radiotherapy in unresectable stage IIIb–IV lung adenocarcinoma, and other independent factors associated with survival in the meantime. The results showed that earlier initiation of local radiotherapy did not prolong overall survival compared with deferred consolidative radiotherapy but significantly prolonged progression-free survival than the deferred one.

Several landmark trials have illustrated the benefit of local consolidative therapy. Ruysscher et al. conducted a prospective single-arm phase II trial to investigate the long-term outcome of adding a radical local treatment to systemic therapy in patients with oligometastatic NSCLC. After following up time for over 7 years, they reached the final analysis. The median overall survival was 13.5 months, and the median progression-free survival was 12.1 months (22, 23). Back then, the major systemic treatment was chemotherapy, and 95% of patients received chemotherapy as part of their front-line treatment in this trial. Gomez et al. conducted a prospective phase II clinical trial on patients with oligometastatic NSCLC, trying to assess the effect of the addition of local consolidative therapy to traditional maintenance therapy. The trial was terminated early due to the substantial efficacy improvement in progression-free survival, from 4.4 to 14.2 months, and overall survival from 17.0 to 41.2 months (15, 16). This finding supports for aggressive local therapy. Another phase II randomized clinical trial compared the efficacy of stereotactic ablative radiotherapy (SBRT) plus chemotherapy versus chemotherapy alone in non-driver gene addicted patients with limited metastatic NSCLC. The results showed a triple PFS in the SBRT-plus arm than chemotherapy alone (17). The trial was also stopped early after an interim analysis due to a significant improvement in PFS. An observational study conducted by Kwint et al. showed a favorable long-term PFS and OS in stage IV NSCLC treated with radical local treatment. The mPFS was 14 months and mOS was 32 months (24). For local consolidative stereotactic ablative radiotherapy (SABR) specifically to intrapulmonary lesions in stage IV NSCLC, the mPFS reached 34.3 months and mOS was not reached (25). Accumulating evidence from clinical trials, research, and translational investigations regarding the potentially curative roles of radiotherapy in advanced NSCLC is converted from palliative ones (26). For patients with unresectable locally advanced NSCLC, Niho et al. released a feasibility study JCOG0402 evaluating the efficacy of gefitinib plus thoracic radiotherapy after induction chemotherapy in 2012 and failed to meet their criterion for feasibility (27). Radiotherapy techniques have evolved over time. In 2020, Xu et al. conducted a retrospective analysis; the results showed favorable survival in the combination of TKIs and radiotherapy with a 6.7% incidence of grade 3 pneumonitis, which is acceptable (28). Another prospective phase II study LOGIK0902 was conducted to evaluate the efficacy and safety of gefitinib induction followed by chemoradiotherapy in EGFR-mutant locally advanced NSCLC. Results showed that the 2-year OS rate reached 90%, with no radiation pneumonitis or treatment-associated death (6). Herein, our results are basically in line with studies mentioned above. Patients who underwent early radiotherapy had significant longer mPFS compared with deferred consolidative radiotherapy, although no significant OS benefit was observed in our analysis for the entire cohort. In addition, patients aged under 60, with a KPS score over 90, systemically treated with combination of TKIs and chemotherapy or bore EGFR 19 mutation are seeing a preferable OS outcome in our analysis, respectively consistent with aforementioned studies.

Referring to the optimal initial time point of radiotherapy, about which clear answers are seldom seen, the present study used ROC to firstly statistically calculate the cutoff time point and performed a statistical analysis on this issue. The optimal initial time point of local radiotherapy was within 53 days after diagnosis, which results in a better mPFS outcome. Of note, patients with newly discovered progression may have developed the progression before their routine checkups during the follow-up process. Thus, the PFS discrepancy between the progression patients and the stable ones should have been slightly larger than we could have detected. For patients harboring TKI-sensitive EGFR mutations, Tang et al. found that the median time to maximal tumor shrinkage was 2 months in EGFR-mutated IIIB or IV NSCLC patients treated with EGFR-TKIs. They suggest local therapy to be adopted during this period (29). Ni et al. found that upfront brain radiotherapy before crizotinib for patients with advanced ALK-positive NSCLC postpones disease progression (30). Analogically, Shafie et al. observed a better intracranial progression-free survival in TKI-treated EGFR/ALK mutant NSCLC treated with early local therapy, regardless of the radiotherapy technique (31). A retrospective analysis observed that the mPFS was 36 months in the LCT plus TKI group and 14 months in the TKI-only group in metastatic NSCLC (32). Similar results were obtained from another retrospective analysis conducted by Xu et al. This study found survival benefit not merely in PFS in patients grouped by treatment modality but also in OS outcome (33). This was a result from stage IV patients. For a larger group including IIIb, the benefits still exist (34). Magnuson et al. conducted a retrospective multi-institutional analysis and found that deferral radiotherapy is associated with inferior OS in patients with EGFR-mutant brain-metastatic NSCLC (35). Wang et al. found a better intracranial progression-free survival (iPFS) but similar OS in upfront intracranial radiation for patients with EGFR-mutant, brain-metastatic NSCLC (36). Similarly, a prolonged time to treatment failure (TTF) and central nervous system progression-free survival (CNS-PFS) for EGFR-mutant NSCLC patients with CNS metastases with upfront brain radiotherapy was found by Saida et al. (37). In our analysis in the EGFR-mutant patient cohort, the PFS of the EAR group is superior to that of the DEF group, but no statistical significance was found in PFS nor in OS between the EAR and DEF groups, possibly because patients with EGFR mutations had better disease control and prognosis than those mutant-free (38), so the initial time point of local intervention had little impact on the overall disease progression. Additionally, and surprisingly, for patients with bone metastasis, early initiation of local radiotherapy is responsible for a preferable OS outcome in our analysis. The principle behind this phenomenon remains to be further explored.

In addition to survival benefits, consideration of toxicities is also a vital aspect when making treatment decisions. Severe adverse events can be a major obstacle to prevent patients from accomplishing a full-course treatment. Previous studies showed the potential increasing risk of toxicities for the combination of radiotherapy with other treatment alternative. Jia et al. reported an increasing risk of radiation pneumonitis in patients with a longer overlap time treated with TKIs and radiotherapy (39). Yet no evident higher occurrence of radiation pneumonitis was observed in the EAR group of our cohort. Quite the reverse, the occurrence of pneumonitis in deferred radiotherapy was higher than that in early-to-initiate radiotherapy in our analysis. The inner relations remain unexplained.

Our analysis has several limitations. Although our robust and detailed datum collection and collation about timing of diagnosis, various treatments, and relapse allowed us to thoroughly evaluate the outcomes of patients in the cohort, the types and extent of treatment patients received varied, and posterior treatment after relapse differed, which could produce unmeasured confounding factors into the subsequent assessment of long-term outcomes. Due to limited conditions, no external dataset was available for any kind of external validation. This results in a high risk of deviation from the cutoff values. Therefore, the conclusion has to be considered carefully and interpreted with caution when guiding doctor conduct. Furthermore, although our cohort represented the largest statistical analysis of optimal initial timing of radiotherapy, it was a selected group of patients with appropriate performance status and comorbidities, the majority of whom underwent first-line radiotherapy, presumably indicating their bipolar conditions of either unbearable local symptoms or physically permitted addition of local therapy. Imbalances in baseline characteristics among sex, M stage, and metastasis sites existed. However, due to the limited number of cases and efforts to avoid loss of available survival data, Cox proportional-hazard analysis was performed, and hazard ratios were calculated to adjust baseline characteristics of the two groups instead of the propensity score matching (PSM) method. In addition, the follow-up time of some patients in our cohort was not long enough for survival data; luckily, the proportion of these patients did not interfere with statistical analysis. Finally, the lack of comparator groups of patients who did not receive any radiation therapy may impede us from distinguishing the true benefit from early radiotherapy.

## Conclusions

In this retrospective single-institution study of 265 patients with stage IIIb–IV unresectable lung adenocarcinoma who underwent front-line local radiotherapy, mOS was 38.6 months and mPFS was 12.7 months. Age >60, bone and brain metastases, multisite metastases, and EGFR 19 mutation were independent predictors associated with OS. The early initiation of local radiotherapy within 53 days after diagnosis resulted in better PFS but no OS outcome. A better OS was observed in patients with bone metastasis who underwent local radiotherapy initiated within 53 days.

## Data Availability Statement

The original contributions presented in the study are included in the article/supplementary material. Further inquiries can be directed to the corresponding authors.

## Ethics Statement

Written informed consent was obtained from the individual(s) for the publication of any potentially identifiable images or data included in this article.

## Author Contributions

XL and XM made substantial contributions to the conception and design of the study. JW and XC helped with data collection and initial phase improvement. XL, ZG, and FT conducted the data. All authors contributed to the article and approved the submitted version.

## Funding

The study received the funding support from the National Natural Science Foundation of China (81627901, 81972863, 81972864, and 82030082) and Natural Science of Shandong Province ZR2020QH177.

## Conflict of Interest

The authors declare that the research was conducted in the absence of any commercial or financial relationships that could be construed as a potential conflict of interest.

## Publisher’s Note

All claims expressed in this article are solely those of the authors and do not necessarily represent those of their affiliated organizations, or those of the publisher, the editors and the reviewers. Any product that may be evaluated in this article, or claim that may be made by its manufacturer, is not guaranteed or endorsed by the publisher.
